# Topological equivalence of stomata distribution patterns across vascular plants

**DOI:** 10.7717/peerj.21152

**Published:** 2026-04-20

**Authors:** Paulette I. Naulin, Sergio A. Estay

**Affiliations:** 1 Departamento de Silvicultura y Conservación de la Naturaleza, Universidad de Chile, Santiago, Chile; 2 Instituto de Ciencias Ambientales y Evolutivas, Universidad Austral de Chile, Valdivia, Chile; 3 Center for Applied Ecology and Sustainability, Facultad de Ciencias Biológicas, Pontificia Universidad Católica de Chile, Santiago, Chile

**Keywords:** Stomata patterning, Minimum spanning tree, Scaling, Leaf architecture, Spatial networks

## Abstract

Stomata are ancient anatomical structures on leaves that regulate the exchange of water vapor, oxygen, and carbon dioxide between plants and the atmosphere. Acting as valve-like gateways between internal tissues and the external environment, stomata may function as locally interacting networks. Theoretical and experimental evidence suggests that local interactions among neighboring stomata influence their function and spatial arrangement. From this perspective, analyzing stomatal distributions as networks may yield novel insights into observed spatial patterns and their generative mechanisms. We hypothesize that variability in stomatal arrangements arises from shared underlying rules, with observed diversity reflecting an epiphenomenon. To test this, we employed a multi-species, two-site approach to assess potential convergences in stomatal distribution. A network-based framework enabled us to reduce individual-level variability and analyze stomatal patterns as interacting systems. Our results show that, across species and environments, stomatal spatial configurations consistently align with a null model linking minimum spanning tree (MST) length to stomatal density. Although a variety of patterns were present, over-dispersed arrangements predominated. These findings suggest that physical constraints during stomatal development could impose limits on the range of viable spatial configurations that can evolve.

## Introduction

Stomata are ancient anatomical structures on leaves that regulate the exchange of gases—water vapor, oxygen, and carbon dioxide—between plants and the atmosphere (Edwards, Kerp & Hass, 1998; Croxdale, 2001; Hetherington & Woodward, 2003; Bergmann, 2004; Matkowski & Daszkowska-Golec, 2023). Traits such as stomatal size, density, shape, and spatial distribution vary widely among individuals, species, and environments. For example, stomatal size (often measured as pore length) has been found to correlate weakly with latitude, although the specific environmental drivers behind this pattern remain unclear (McKown et al., 2014). Results from common garden experiments suggest that pore length may co-vary with climatic conditions (Carlson, Adams & Holsinger, 2016). These findings support the idea that stomatal traits exhibit a degree of plasticity, with potentially important implications for plant physiological performance (Drake, Froend & Franks, 2013).

Stomatal density is the simplest and most commonly used descriptor of their spatial distribution on the leaf surface. For instance, McKown et al. (2014) reported that stomatal density in *Populus* species is negatively correlated with both temperature and precipitation across a latitudinal gradient. In contrast, Fontana et al. (2017) observed a slightly positive relationship between stomatal density and rainfall in *Salix miyabeana*, highlighting the complexity of environmental influences. Beyond temperature and precipitation, other factors also appear to modulate stomatal density. Almeida Filho, Machicao & Bruno (2017) found that the distance between neighboring stomata, likely influenced by overall density, increased with light exposure, suggesting that light availability can also shape stomatal spatial patterns.

Stomatal spatial arrangement has traditionally been described as uniform, with distinct “stomata-free” regions surrounding each stoma (Sachs, 1991). However, Naulin, Valenzuela & Estay (2017) demonstrated that this perception may be an artifact of the point-pattern analysis methods commonly employed in such studies. By applying a more realistic disc-pattern analysis, they showed that stomatal distributions can span the full theoretical spectrum—from uniform to random to clustered patterns. For example, Fanourakis, Heuvelink & Carvalho (2015) found that stomata were closer together than the expected in *Rosa hybrida,* even under different humidity regimes, suggesting that in some cases stomatal function (in this case affected by high relative air humidity) was unrelated to the observed spatial pattern. To fully understand stomatal organization and its underlying mechanisms, spatial patterning must be explicitly analyzed. Various biological (*e.g.*, genetic) and physico-chemical mechanisms, such as activation–inhibition processes, appear to regulate stomatal spacing to avoid configurations that impair stomatal function (Serna & Fenoll, 1997; Illian et al., 2008; Dow, Berry & Bergmann, 2014; Fanourakis, Heuvelink & Carvalho, 2015; Fiorin, Brodribb & Anfodillo, 2016). Thus, stomatal distribution can be considered an anatomical trait subject to strong ecological and evolutionary pressures (Fiorin, Brodribb & Anfodillo, 2016; Xiong & Flexas, 2020).

Evidence from common garden experiments indicates that intraspecific variation in stomatal density can be adaptive. For example, Carlson, Adams & Holsinger (2016) identified a positive selection gradient favoring individuals with stomatal traits suited to drier and hotter environments. At the interspecific level, Yang, Chen & Xian (2023) analyzed 90 species and found that, depending on the spatial scale, both aggregated and uniform (*i.e.,* over-dispersed) stomatal distributions exhibit weak phylogenetic signals, suggesting a partial influence of evolutionary history. While these studies offer valuable insights, the relative contributions of evolutionary and environmental factors in shaping stomatal density and distribution remain unclear. Zhang et al. (2012), for instance, reported species-specific responses: in some species, stomatal traits were predominantly shaped by environmental conditions, whereas in others, genetic control was more pronounced.

The spatial and temporal context in which a leaf develops plays a crucial role in shaping stomatal density and distribution. Even today, variation in these traits is used to infer past environmental conditions, particularly in palaeobotanical records (*e.g.*, Uhl & Kerp, 2005). Conceptually, stomata function as windows that connect internal leaf tissues with the external environment. Each stoma can be thought of as a valve-like structure that regulates gas exchange within a specific region of the leaf surface (Clark et al., 2022). In particular, Watts et al. (2024) pointed to the relationship between the stomatal size and the associated mesophyll volume it supplies with CO_2_. In this way, we can think of stomata as a spatial network representing how gases diffuse through the leaf tissues. Both theoretical and experimental studies have shown that stomatal function is influenced by neighboring stomata (Haefner, Buckley & Mott, 1997; Mott, Denne & Powell, 1997). These local interactions suggest that stomatal arrangements operate as locally interacting networks. If this is the case, analyzing stomatal distributions and their structural properties through a network-based framework could offer novel insights into the spatial patterns observed and the underlying generative mechanisms. We therefore hypothesize that the apparent variability in stomatal distributions arises from shared generative rules, and that the diversity of observed patterns may simply be an epiphenomenon.

In this study, we apply a multi-species, two-site approach to identify potential commonalities in observed stomatal distribution patterns. By using a network-based framework, we minimize the confounding effects of individual variability and treat stomatal arrangements as interacting biological systems.

### Methods

Portions of this text were previously published as part of a preprint (Naulin & Estay, 2025).

### Biological data

Sixty-six plant species, spanning four classes, 27 orders, and 41 families, were sampled from two arboreta: the Antumapu Arboretum (hereafter Antumapu; 33.57°S, 70.63°W) and the Frutillar Arboretum (hereafter Frutillar; 41.12°S, 73.03°W), both managed by the Faculty of Forestry and Nature Conservation, University of Chile (Table S1). These sites are approximately 980 km apart. Antumapu is located in a warm-summer Mediterranean climate (Köppen classification: Csb), with average summer and winter temperatures of 19 °C and 9.5 °C, respectively, and an annual precipitation of 371 mm (Santibáñez, 2017). In contrast, Frutillar is situated in southern Chile within a temperate oceanic climate (Köppen classification: Cfb), characterized by average summer and winter temperatures of 14.3 °C and 7 °C, respectively, and an annual precipitation of 1,516 mm (Santibáñez, 2017).

For each species, three fully developed leaves were collected. Leaves were cleared following the protocol described by Castellaro et al. (2007) and mounted on glass slides for microscopic examination and imaging. In this last step, 16 samples were destroyed during processing and finally 180 samples were available for analysis. Anatomical measurements were performed using images of approximately one mm^2^ from the central third of the abaxial leaf surface. The dataset includes hypostomatous and amphistomatous species. To ensure comparability of leaf structural features among phylogenetically and ecologically diverse taxa, all measurements were consistently obtained from the abaxial leaf surface. This standardized approach follows common practice in anatomical leaf studies. For size measurements, photographs were taken at 40x magnification, and for stomatal density and distribution, at 10x magnification.

The selection of the area to be sampled for density was performed by observing the histological sample and selecting a representative area without damage or folds. A macro lens was then used to cut out a one mm^2^ area, which served as the sampling area. To estimate spatial properties, the centroid of each stomatal complex was determined using Cartesian coordinates in ImageJ software (Rasband, 1997; Schneider, Rasband & Eliceiri, 2012). For each sample, the total number of stomata was recorded. Additionally, the length and width of 30 stomata were measured per leaf. If the number of stomata in the sampled area was less than 30, the search area, centered in the original one mm^2^ area, was increased until the sample was complete (Table S1). From now on, we will refer to the average of the length and width as the stomatal diameter.

### Network reconstruction

To represent potential local interactions, we connected neighboring polygons by linking the centroids of adjacent stomata. This process generates a Delaunay triangulation—the dual graph of the Voronoi diagram—which connects each stoma to its nearest neighbors, forming a dense network in which each node (stoma) typically has a degree of approximately six (Aurenhammer, Klein & Lee, 2013).

Within the Delaunay triangulation lies a key subgraph known as the Minimum Spanning Tree (MST). The MST is a loop-free graph that connects all nodes while minimizing the total edge length (Prim, 1957). In the context of complex spatial patterns, the MST is often used to characterize dominant connectivity structures. The MST has a long history of applications in biology and ecology, and its topological properties are well-documented (Dussert et al., 1987; Dussert et al., 1989; Cantwell & Forman, 1993; Jones, Lonergan & Mainwaring, 1996; Wallet & Dussert, 1997; Bunn, Urban & Keitt, 2000; Urban & Keitt, 2001; Dry, Preiss & Wagemans, 2012). More recently, Liu et al. (2021) applied MST-based methods to the analysis of stomatal arrangements, making it an ideal starting point for identifying common structural features of stomatal distribution across plant species—particularly in light of the trade-off between epidermal space use for photosynthesis and water conservation (Lawson & McElwain, 2016).

MSTs exhibit a range of topological properties that can be used to classify spatial arrangements of objects. Metrics such as total MST length, mean edge length, and the standard deviation of edge lengths are commonly used to characterize spatial configurations (Hoffman & Jain, 1983; Dussert et al., 1987). Under the assumption of complete spatial randomness (*i.e.,* a Poisson process) in two dimensions, the expected MST length approaches the following limit:

(1) \begin{eqnarray*}{L}_{mts}=\beta (N\ast A)^{1/2}\end{eqnarray*}

where N is the number of points (stomata), A is the area of observation, and β is a constant that depends on the spatial structure of the distribution. Empirical estimates place β between 0.63 and 0.64 under random conditions (Beardwood, Halton & Hammersley, 1959; Bertsimas & Ryzin, 1990; Avram & Bertsimas, 1992; Jaillet, 1993; Cortina-Borja & Robinson, 2000). Values of β lower than this range indicate clustering, while higher values suggest over-dispersion.

In this study, we calculated the MST for each sample as a representation of the stomatal network. For each MST, we measured the total length and evaluated deviations from the null model (Eq. 1) to infer differences in spatial arrangement.

To contextualize each sample, we compared its MST length to three simulated benchmark patterns: clustered, random (Poisson), and over-dispersed. Simulated patterns used the same number of stomata and observation area as the empirical sample. Clustered patterns were generated using four spatial clusters, while over-dispersed patterns applied a minimum inhibition distance equal to the stomatal diameter of the sample. Each scenario was simulated 1,000 times.

All analyses were conducted in R (2024), using the packages stpp, netgen, and emstree (Gabriel, Rowlingson & Diggle, 2013; Bossek, 2024; Quadros, 2025).

## Results

**Figure 1:**
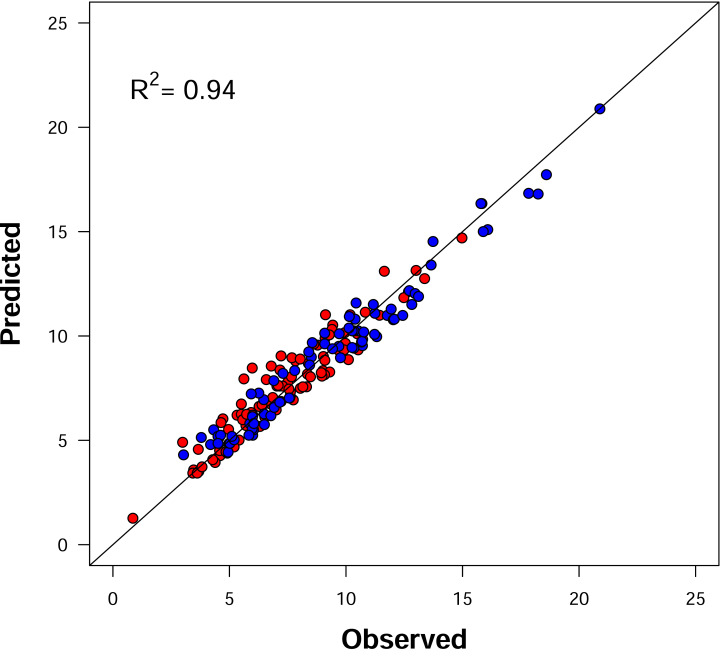
Relationship between observed and predicted MST lengths across all samples. MST length was rescaled to millimeters. Each point represents a sample, with blue indicating Antumapu and red indicating Frutillar. The diagonal line denotes the 1:1 relationship, representing a perfect fit between observed and predicted values based on the theoretical model.

Stomatal density across samples ranged from three to 777 stomata per mm^2^. The lowest density was observed in a sample of the fern *Asplenium trilobum* (Aspleniaceae), while the highest was recorded in *Cryptocarya alba* (Lauraceae). In general, higher stomatal densities were found in samples from the Mediterranean site (Antumapu), whereas stomatal diameter showed no consistent pattern between the two sites (Fig. 1; Table S2).

The same samples that exhibited the lowest and highest stomatal densities also showed the minimum and maximum MST lengths, with values ranging from 858 µm to 20,894 µm. As expected, there was a strong positive correlation between the number of stomata per sample and the total MST length (Pearson’s *r* = 0.945; 95% CI [0.927–0.959]). Additionally, stomatal number was significantly negatively correlated with average stomatal diameter (*r* = −0.568; 95% CI [−0.659 to −0.459]).

Despite the three orders of magnitude variation in stomatal density, MST length across all samples was accurately predicted as a function of stomatal density, with a high coefficient of determination (*R*^2^ = 0.94; Fig. 1). However, the estimated value of the scaling parameter β was significantly higher than expected under complete spatial randomness. Specifically, we obtained a mean β of 0.7492 (95% CI [0.7393–0.7591]), which exceeds the typical range reported for Poisson-distributed patterns (0.63–0.64), suggesting a consistent tendency toward over-dispersion in stomatal spatial arrangements.

When comparing observed MST lengths against simulated distributions, 2.8% of the samples (five out of 180) were classified as clustered, 27.2% (49 samples) as random, and 32.8% (59 samples) as over-dispersed. A total of 49.4% of the samples (89 samples) did not fall exclusively within any single category. This is due to overlapping confidence intervals, which allowed some samples to be classified into multiple categories.

Among the unclassified samples, 10% (18 samples) fell between the confidence intervals of the random and over-dispersed simulations, while 39.4% (71 samples) exceeded the upper limit of the over-dispersed range. In total, 72% of the samples (130 out of 180) either fell within or above the over-dispersed range, and 82% (148 samples) fell above the random distribution range.

**Figure 2:**
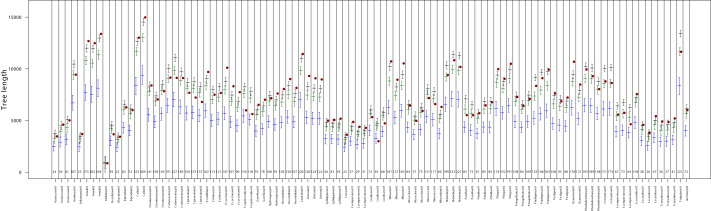
Comparison of observed MST length values (red points) with simulated distributions under clustered (blue), random (green), and over-dispersed (black) spatial scenarios for each sample from the Frutillar site. The *x*-axis shows sample identifiers, with the number of stomata in each sample indicated by small numbers above the axis. Bars represent the median and 95% confidence interval of the MST length from 1,000 simulations per spatial pattern scenario described in the Methods section.

**Figure 3:**
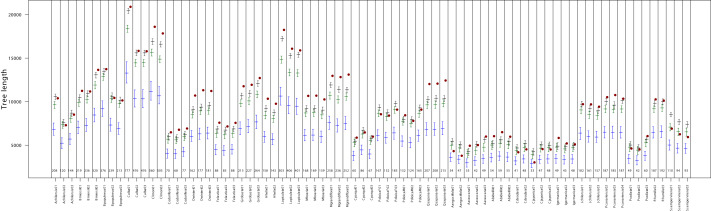
Comparison of observed MST length values (red points) with simulated distributions under clustered (blue), random (green), and over-dispersed (black) spatial scenarios for each sample from the Antumapu site. The *x*-axis shows sample identifiers, with the number of stomata in each sample indicated by small numbers above the axis. Bars represent the median and 95% confidence interval of the MST length from 1,000 simulations per spatial pattern scenario described in the Methods section.

No clear taxonomic pattern was observed in relation to the type of spatial arrangement. Full classification results for each sample are provided in Figs. 2 and 3 and Table S2.

## Discussion

Trade-offs and relationships among stomatal size, density, and spatial arrangement have garnered increasing attention over the past decade (*e.g.*, Beerling & Franks, 2010; Brodribb et al., 2014). The diverse patterns reported in the literature appear to result from an incomplete understanding of the interplay of genetic and environmental factors (Zhang et al., 2012). In this study, we collected data from a broad range of species growing in two ecologically contrasting environments. The stomatal density values observed in our samples were consistent with previous findings (Franks & Beerling, 2009; Haworth et al., 2023). Notably, for the species present in both sites the highest stomatal density and MST length were recorded in samples from the Mediterranean site (Antumapu) (Fig. 1), supporting the hypothesis that increased stomatal density under higher temperatures may represent an adaptive strategy to enhance gas exchange and evaporative cooling (Hill et al., 2015).

The strong positive correlation between stomatal density and MST length aligns with theoretical expectations from our model (Eq. 1): a greater number of nodes necessarily results in a longer minimum spanning tree (Beardwood, Halton & Hammersley, 1959; Cortina-Borja & Robinson, 2000). Furthermore, our findings are consistent with prior studies reporting a significant negative correlation between stomatal density and stomatal size across species (Franks & Beerling, 2009; Haworth et al., 2023).

However, when we focused on the subset of species present at both sites, we observed a more nuanced pattern (Table S2). With only one exception (*A. chilensis*), all species exhibited higher stomatal densities in the Mediterranean climate (Antumapu) compared to the temperate oceanic climate (Frutillar). In contrast, stomatal size did not follow a consistent trend across environments. These results suggest that, at the intraspecific level, stomatal density may be more responsive to environmental conditions than stomatal size—indicating a greater degree of plasticity. Nevertheless, this interpretation is based on a limited sample of seven species, and further studies with larger species pools are needed to confirm this pattern.

Although our dataset includes numerous species from diverse biogeographical origins and two ecologically distinct environments, the spatial configuration of stomata across all samples was well-described by the theoretical model (Eq. 1; Fig. 1). The strong fit of this model suggests that, across a wide range of lineages and taxonomic groups, stomatal arrangements adhere to a shared generative rule—one in which MST length scales with the square root of stomatal density and a constant parameter (β) representing spatial inhibition or repulsion among stomata. This result implies a form of topological equivalence across species and environments. In this case at least a pair of subgraphs that can be mapped reciprocally between species’ MST. Also, the observed spatial patterns can be interpreted as scale transformations—shrinking or stretching—of a common underlying configuration.

At the spatial resolution of our analysis (∼one mm^2^), no major deviations from this model were observed between species or sites. The estimated β value (>0.74) points to consistent repulsion or inhibition among stomata, aligning with both the “one-cell spacing” rule (Lawson & McElwain, 2016) and the finite-size constraint imposed by the physical dimensions of stomata themselves (Naulin, Valenzuela & Estay, 2017). Our simulation results further support this interpretation. Although we examined a limited set of point process scenarios (single configurations for both clustered and over-dispersed patterns), the majority of samples (72%) exhibited levels of over-dispersion equal to or greater than those generated by the over-dispersed simulations (Figs. 2 and 3).

As previously discussed, two main components likely contribute to this over-dispersion. First, stomata are not point-like objects—their finite size prevents overlap and inherently induces spatial repulsion (Naulin, Valenzuela & Estay, 2017). Since our simulations are based on idealized point processes, part of the observed over-dispersion likely reflects this geometric constraint. Second, and more importantly, 39% of samples displayed levels of over-dispersion that exceeded our simulation range even after accounting for stomatal size. This suggests the presence of active biological mechanisms. For example, the necessary interaction between guard cells and surrounding cells that allows the opening and closing of stomata is one explanation for the one-cell-spacing rule. Also, genetic mechanisms enforce spatial inhibition during stomatal development, further supporting the view that stomatal patterns are shaped by endogenous regulatory processes in addition to biophysical constraints.

If our model accurately reflects the mechanisms governing stomatal spatial organization, it carries several important implications—both quantitative and qualitative—for understanding how stomata can be distributed on the epidermis. At low densities, there are potentially thousands of viable spatial configurations that achieve a functional balance in the trade-off between maximizing photosynthetic area and minimizing water loss. However, evolutionary increases in stomatal density not only force reductions in stomatal size (Franks & Beerling, 2009; Haworth et al., 2023), but also dramatically constrain the number of feasible spatial arrangements. Beyond a certain threshold, only over-dispersed configurations remain viable (Feder, 1980; Parisi & Zamponi, 2010).

This constraint arises from fundamental geometric principles: the number of possible arrangements of finite-sized objects in a fixed area is far more limited than for point-like objects (Simberloff, 1979; Wu et al., 1987). As stomata cannot be infinitely small, increasing their number while preserving function requires increasingly regular spacing. In the theoretical limit, this constraint leads to a near-regular grid-like distribution.

It is important to note that these findings apply specifically to the spatial scale analyzed in this study (∼one mm^2^). At broader spatial scales, additional structural constraints—such as those imposed by leaf venation networks—may further shape or override local stomatal arrangements (Matos et al., 2025). Future studies incorporating multiscale analyses and biomechanical modeling may provide deeper insights into how such hierarchical constraints interact across spatial levels.

Stomatal size and density are key determinants of CO_2_ conductance in leaves (Franks & Beerling, 2009) and are therefore subject to strong selective pressures to balance mesophyll demand for carbon assimilation with efficient water vapor diffusion (Lawson & McElwain, 2016; Haworth et al., 2023). In this sense, Watts et al. (2024) provides clear evidence that each stomata is associated with a proportional area in the mesophyll, which supports our conclusion that at high stomatal densities, only regular spacing is possible. Our results suggest that further increases in stomatal density are only feasible through heightened over-dispersion, progressively driving stomatal arrangements toward grid-like spatial patterns across the leaf surface.

## Supplemental Information

10.7717/peerj.21152/supp-1 Supplemental Information 1 List of plant species and corresponding samples N indicates the number of samples analyzed per species. Code refers to the identifier used throughout the text and figures.

10.7717/peerj.21152/supp-2 Supplemental Information 2 Summary of results for each analyzed sample N stomata refers to the total number of stomata identified in the sample. Sampled area indicates the area (in mm^2^) of the microscopic field used for measurements. Site refers to the sampling location: A = Antumapu, F = Frutillar. See Methods for further details on sample processing and measurements.

## Additional Information and Declarations

### Data Availability

The following information was supplied regarding data availability: The raw data are available in the Supplemental Files.
