# Supplementary material for: Topological equivalence of stomata distribution patterns across vascular plants
Source: PeerJ. 2026 Apr 20;14:e21152. doi: 10.7717/peerj.21152 (PMC13105184; doi:10.7717/peerj.21152)
Supplement: Supplemental Information 1 — N indicates the number of samples analyzed per species. Code refers to the identifier used throughout the text and figures. [file peerj-14-21152-s001.pdf]

Table S1. List of plant species and corresponding samples used in this study. N indicates the number of samples analyzed per species. Code refers to the identifier used throughout the text and figures.

Code	Species	class	order	family	N Frutillar	N Antumapu	N total
Aangustifolia	Araucaria angustifolia (Bertol.) Kuntze	Pinopsida	Pinales	Araucariaceae	2	0	2
Aaraucana	Araucaria araucana (Molina) K.Koch	Pinopsida	Pinales	Araucariaceae	3	3	6
Abidwilli	Araucaria bidwillii Hook.	Pinopsida	Pinales	Araucariaceae	3	0	3
Achilensis	Aristotelia chilensis (Molina) Stuntz	Magnoliopsida	Oxalidales	Elaeocarpaceae	3	1	4
Adiantumsp	Adiantum L.	Polypodiopsida	Polypodiales	Pteridaceae	0	1	1
Ameli	Amomyrtus meli (Phil.) D.Legrand & Kausel	Magnoliopsida	Myrtales	Myrtaceae	0	3	3
Atriloba	Asplenium trilobum Cav.	Polypodiopsida	Polypodiales	Aspleniaceae	0	1	1
Bcordatum	Blechnum cordatum (Desv.) Hieron.	Polypodiopsida	Polypodiales	Blechnaceae	0	1	1
Bhastatum	Blechnum hastatum Kaulf.	Polypodiopsida	Polypodiales	Blechnaceae	0	1	1
Bmiersii	Beilschmiedia miersii (Gay) Kosterm.	Magnoliopsida	Laurales	Lauraceae	3	0	3
Bpendula	Betula pendula Roth	Magnoliopsida	Fagales	Betulaceae	0	2	2
Bpopulneus	Brachychiton populneus (Schott & Endl.) R.Br.	Magnoliopsida	Malvales	Malvaceae	3	0	3
Calba	Cryptocarya alba (Molina) Looser	Magnoliopsida	Laurales	Lauraceae	3	2	5
Cdeodara	Cedrus deodara (Lamb.) G.Don	Pinopsida	Pinales	Pinaceae	2	0	2
Chookeranum	Crinodendron hookerianum Gay	Magnoliopsida	Oxalidales	Elaeocarpaceae	0	3	3
Cjaponica	Camellia japonica L.	Magnoliopsida	Ericales	Theaceae	3	0	3
Climon	Citrus ×limon (L.) Osbeck	Magnoliopsida	Sapindales	Rutaceae	2	0	2
Codorifera	Colliguaja odorifera Molina	Magnoliopsida	Malpighiales	Euphorbiaceae	3	0	3
Cotoneaster	Cotoneaster Medik.	Magnoliopsida	Rosales	Rosaceae	0	3	3
Cpaniculata	Caldcluvia paniculata (Cav.) D.Don	Magnoliopsida	Oxalidales	Cunoniaceae	0	3	3
Dwinteri	Drimys winteri J.R.Forst. & G.Forst.	Magnoliopsida	Canellales	Winteraceae	3	0	3
Echilensis	Elytropus chilensis (A.DC.) Müll.Arg.	Magnoliopsida	Gentianales	Apocynaceae	0	2	2
Ecoccineum	Embothrium coccineum J.R.Forst. & G.Forst.	Magnoliopsida	Proteales	Proteaceae	0	3	3
Ecordifolia	Eucryphia cordifolia Cav.	Magnoliopsida	Oxalidales	Cunoniaceae	0	1	1
Fcupressoides	Fitzroya cupressoides (Molina) I.M.Johnst.	Pinopsida	Pinales	Cupressaceae	0	2	2
Felastica	Ficus elastica Roxb.	Magnoliopsida	Rosales	Moraceae	3	0	3
Gavellana	Gevuina avellana Molina	Magnoliopsida	Proteales	Proteaceae	0	2	2
Grobusta	Grevillea robusta A.Cunn. ex R.Br.	Magnoliopsida	Proteales	Proteaceae	3	0	3
Hhelix	Hedera helix L.	Magnoliopsida	Apiales	Araliaceae	2	0	2
Hserratifolia	Hydrangea serratifolia (Hook. & Arn.) F.Phil.	Magnoliopsida	Cornales	Hydrangeaceae	0	3	3
Hydrangea	Hydrangea Gronov. ex L.	Magnoliopsida	Cornales	Hydrangeaceae	0	2	2
Igermanica	Iris ×germanica L.	Liliopsida	Asparagales	Iridaceae	3	0	3
Jchilensis	Jubaea chilensis (Molina) Baill.	Liliopsida	Arecales	Arecaceae	3	0	3
Lapiculata	Luma apiculata (DC.) Burret	Magnoliopsida	Myrtales	Myrtaceae	3	1	4
Lhirsuta	Lomatia hirsuta (Lam.) Diels	Magnoliopsida	Proteales	Proteaceae	0	3	3
Lphilippiana	Laurelia philippiana Looser	Magnoliopsida	Laurales	Atherospermataceae	0	3	3
Lradicans	Luzuriaga radicans Ruiz & Pav.	Liliopsida	Liliales	Alstroemeriaceae	0	3	3
Lrosea	Lapageria rosea Ruiz & Pav.	Liliopsida	Liliales	Philesiaceae	0	1	1
Lsempervirens	Laurelia sempervirens (Ruiz & Pav.) Tul.	Magnoliopsida	Laurales	Atherospermataceae	0	3	3
Mboaria	Maytenus boaria Molina	Magnoliopsida	Celastrales	Celastraceae	3	3	6
Mcoccinea	Mitraria coccinea Cav.	Magnoliopsida	Lamiales	Gesneriaceae	0	3	3

Mexsucca	Myrceugenia exsucca (DC.) O.Berg	Magnoliopsida	Myrtales	Myrtaceae	0	2	2
Mgrandiflora	Magnolia grandiflora L.	Magnoliopsida	Magnoliales	Magnoliaceae	3	0	3
Muehlenbeckia	Muehlenbeckia hastulata (J.E.Sm.) I.M.Johnst.	Magnoliopsida	Caryophyllales	Polygonaceae	0	1	1
Ndombeyii	Nothofagus dombeyi (Mirb.) Oerst.	Magnoliopsida	Fagales	Nothofagaceae	0	3	3
Palqui_	Cestrum parqui (Lam.) L'Hér.	Magnoliopsida	Solanales	Solanaceae	3	0	3
Pandina	Prumnopitys andina (Poepp. ex Endl.) de Laub.	Pinopsida	Pinales	Podocarpaceae	0	2	2
Pboldus	Peumus boldus Molina	Magnoliopsida	Laurales	Monimiaceae	0	3	3
Pcanariensis	Phoenix canariensis H.Wildpret	Liliopsida	Arecales	Arecaceae	3	0	3
Pdioicah	Phytolacca dioica L.	Magnoliopsida	Caryophyllales	Phytolaccaceae	3	0	3
Pdioicam	Phytolacca dioica L.	Magnoliopsida	Caryophyllales	Phytolaccaceae	3	0	3
Plingue	Persea lingue (Ruiz & Pav.) Nees	Magnoliopsida	Laurales	Lauraceae	0	3	3
Pmagellanica	Philesia magellanica J.F.Gmel.	Liliopsida	Liliales	Philesiaceae	0	3	3
Pnubigenus	Podocarpus nubigenus Lindl.	Pinopsida	Pinales	Podocarpaceae	0	3	3
Pradiata	Pinus radiata D.Don	Pinopsida	Pinales	Pinaceae	3	0	3
Psaligna	Podocarpus salignus D.Don	Pinopsida	Pinales	Podocarpaceae	0	3	3
Qsaponaria	Quillaja saponaria Molina	Magnoliopsida	Fabales	Quillajaceae	3	2	5
Rhododendron	Rhododendron L.	Magnoliopsida	Ericales	Ericaceae	0	3	3
Rhumilis	Raphia humilis A.Chev.	Liliopsida	Arecales	Arecaceae	2	0	2
Scassioides	Sophora cassioides (F.Phil.) Sparre	Magnoliopsida	Fabales	Fabaceae	0	1	1
Sconspicua	Saxegothaea conspicua Lindl.	Pinopsida	Pinales	Podocarpaceae	0	2	2
Sscandens	Sarmienta scandens (Brandis) Pers.	Magnoliopsida	Lamiales	Gesneriaceae	0	3	3
Ssempervirens	Sequoia sempervirens (D.Don) Endl.	Pinopsida	Pinales	Cupressaceae	3	3	6
Tcorymbosus	Tristerix corymbosus (L.) Kuijt	Magnoliopsida	Santalales	Loranthaceae	0	3	3
Tstipularis	Tepualia stipularis (Hook.fil.) Griseb.	Magnoliopsida	Myrtales	Myrtaceae	0	1	1
Uncinia	Uncinia Pers.	Liliopsida	Poales	Cyperaceae	0	1	1
**Total**					**79**	**101**	**180**
