# Supplementary material for: Topological equivalence of stomata distribution patterns across vascular plants
Source: PeerJ. 2026 Apr 20;14:e21152. doi: 10.7717/peerj.21152 (PMC13105184; doi:10.7717/peerj.21152)
Supplement: Supplemental Information 2 — N stomata refers to the total number of stomata identified in the sample. Sampled area indicates the area (in mm2) of the microscopic field used for measurements. Site refers to the sampling location: A = Antumapu, F = Frutillar. See Methods for further details on sample processing and measurements. [file peerj-14-21152-s002.pdf]

Table S2. Summary of results for each analyzed sample. N stomata refers to the total number of stomata identified in the sample. Sampled area indicates the area (in mm²) of the microscopic field used for measurements. Site refers to the sampling location: A = Antumapu, F = Frutillar. See Methods for further details on sample processing and measurements.

						Confidence intervals for simulations					
						Clustered simulations		Random simulations		Over-dispersed simulations	
Sample Code	N stomata	MST length (µm)	Stoma diameter (µm)	Sampled area (mm^2)	Site	95% Low	95% Upper	95% Low	95% Upper	95% Low	95% Upper
Aangustifolia1	54	4323.9	52.19	1.000	A	3128.9	4118.5	4552.3	5444.5	5094	5769.8
Aangustifolia2	47	3793.1	51.06	1.000	A	2911.4	3838.1	4214.9	5120.1	4635.6	5383.1
Aaraucana1	24	3464.6	73.39	0.953	F	2043.6	2950.6	2800	3730.4	3283.8	4079.8
Aaraucana2	34	4596.7	72.62	0.953	F	2432.2	3350.3	3411.6	4333	4115.6	4896.8
Aaraucana3	41	5006.6	75.03	0.953	F	2640.6	3632.3	3843.5	4713.6	4712	5414.6
Aaraucana1	35	4942.5	49.11	1.000	A	2492	3438.6	3580.9	4501.5	3830.9	4652.2
Aaraucana2	42	5018.6	78.50	1.000	A	2709.9	3669.1	3926.7	4829.3	4776.1	5427.5
Aaraucana3	49	5997.5	79.39	1.000	A	2930.9	3901.5	4321.4	5224.9	5351.2	5928.6
Abidwilli1	54	6038.6	31.66	1.000	A	3077.6	4087.6	4508.2	5451.1	4748.9	5526.6
Abidwilli2	59	6500.8	44.41	1.000	A	3260.9	4248.2	4771	5650.6	5204.3	5962.3
Abidwilli3	55	5979.2	45.70	1.000	A	3125.6	4139.6	4590.5	5481.3	5008.2	5742.3
Achilensis1	207	9432.3	21.44	0.953	F	6001	7437.6	8989.4	9812.8	10079.8	10811.2
Achilensis1	208	10391.3	27.30	1.000	A	6151.8	7549.8	9213	10021.4	10250.8	10882.2
Achilensis2	120	7297.2	17.90	1.000	A	4610.6	5856	6941.9	7750.7	7158.3	7976.3
Achilensis3	144	8513.2	28.70	1.000	A	5098.3	6331	7642.9	8479.7	8298.2	9021
Adiantumsp1	23	3685.8	32.28	0.953	F	2008.8	2860	2735.9	3639.9	2908.3	3861.6
Ameli1	275	12682.6	18.41	0.953	F	6946	8458.7	10406.5	11196.4	11610.4	12327.7
Ameli2	262	12481.6	22.36	0.953	F	6768	8309.2	10128	10942.8	11599.2	12289.8
Ameli3	304	13373.6	21.99	0.953	F	7303.7	8922.3	10929.2	11740.8	12617.5	13279.6

Atriloba1	3	858.2	61.69	0.953	F	NA	NA	290	1450.1	373.9	1535.7
Bcordatum1	39	3664.2	34.34	0.953	F	2544.5	3500.2	3737.6	4595.2	4033.2	4927.8
Bhastatum1	22	3428.6	40.81	0.953	F	1979.5	2833.7	2614.7	3599.6	2873.1	3804
Bmiersii1	219	11241.8	19.70	1.000	A	6322.8	7775.1	9453.1	10298.6	10047.9	10790.2
Bmiersii2	236	11173.1	20.60	1.000	A	6594.2	8052.5	9849.7	10666.2	10571.7	11254.5
Bmiersii3	320	13652.6	22.00	1.000	A	7678.3	9259.3	11477.1	12311	12720.3	13375.7
Bpendula1	82	6271.1	27.87	0.953	F	3769.3	4809.8	5585.4	6387.6	6145	6932.8
Bpendula2	72	6000.5	28.51	0.953	F	3514.6	4541.9	5178.9	6038.1	5706.5	6516.1
Bpopulneus1	376	13735.3	13.70	1.000	A	8315.2	10093.2	12457.5	13257.1	13097.7	13822.6
Bpopulneus2	239	10433.8	13.00	1.000	A	6622.8	8118	9914.5	10706.7	10211.4	11004.1
Bpopulneus3	213	10143.1	17.10	1.000	A	6263.9	7573.8	9355.4	10178.7	9801	10520.2
Calba1	323	13008.1	12.30	0.953	F	7517.3	9178.4	11303.7	12060.9	12246.8	13015
Calba2	404	14975.8	15.82	0.953	F	8496.9	10291.7	12632.6	13412.5	14178	14884.4
Calba1	777	20894.0	15.00	1.000	A	12147.6	14573.9	17966.8	18783.4	20129.7	20726.9
Calba2	476	15832.0	16.00	1.000	A	9390.1	11351.1	14054.7	14841.3	15283.4	15924.3
Calba3	476	15790.1	16.00	1.000	A	9315.1	11368	14032	14835.4	15273	15901.1
Cdeodara1	41	4190.3	52.17	1.000	A	2749.8	3670.4	3928.9	4814.6	4287.6	5059.9
Cdeodara2	48	4525.1	57.40	1.000	A	2955.4	3890.2	4250.6	5158.2	4816.6	5539.4
Chookeranum1	141	8387.9	13.10	0.953	F	4922.2	6150.2	7408.6	8216.1	7886.9	8735.3
Chookeranum2	108	7037.4	18.80	0.953	F	4309.1	5433	6438	7234	6964.5	7757
Chookeranum3	145	7833.1	13.95	0.953	F	5019	6234.3	7490	8335.9	8022.6	8874.3
Cjaponica1	33	3030.5	62.85	1.000	A	2429.7	3346.2	3440.4	4367.9	3850.4	4623.5
Cjaponica2	47	4556.6	50.36	1.000	A	2919.9	3855.1	4217.8	5112	4637.2	5379.4
Cjaponica3	48	4509.2	41.10	1.000	A	2947.5	3927.8	4241.6	5174.1	4593.3	5372
Climon1	560	18599.9	14.90	1.000	A	10185.7	12335.4	15244.7	16046.2	16566.8	17229.4
Climon2	505	17837.9	19.00	1.000	A	9745.3	11730.5	14496.7	15258.1	16278.5	16882.5

Codorifera1	70	6484.1	34.20	1.000	A	3520.9	4565.5	5236.6	6108.8	5595.1	6374.9
Codorifera2	68	6778.8	31.30	1.000	A	3495.6	4523.2	5177.2	6000.8	5439.4	6199.9
Codorifera3	77	6920.7	29.90	1.000	A	3725.8	4770.3	5537.3	6387.2	5804.2	6593.3
Cotoneaster1	191	9152.3	22.02	0.953	F	5765.2	7125	8653.9	9423.1	9664.1	10398.5
Cotoneaster2	227	9120.3	22.79	0.953	F	6310.9	7736.7	9418.7	10246.1	10709.2	11422.5
Cotoneaster3	183	9104.8	20.33	0.953	F	5642	6980.5	8430.9	9246.1	9337.3	10084.3
Cpaniculata1	150	7679.9	16.68	0.953	F	5042.8	6351.4	7636.5	8437.3	8258.4	9076.6
Cpaniculata2	153	7214.4	17.99	0.953	F	5158.6	6399.2	7724.2	8509.3	8389.6	9151.7
Cpaniculata3	137	6793.5	15.83	0.953	F	4881.1	6052.7	7268.2	8120.7	7841.5	8651.2
Dwinteri1	162	10705.5	25.00	1.000	A	5444.3	6689.9	8126.7	8952.7	8720.8	9437.7
Dwinteri2	177	11317.9	20.80	1.000	A	5667	6967.5	8525.9	9328.8	9016.7	9728.3
Dwinteri3	181	11227.3	23.00	1.000	A	5721.3	7082.2	8561.3	9401.5	9220.5	9925.1
Echilensis1	113	7496.2	36.21	0.953	F	4442.8	5573.2	6612.4	7402.7	7674.7	8329.8
Echilensis2	121	7661.3	34.30	0.953	F	4578.1	5752.6	6830.3	7651.7	7895.3	8576.7
Ecoccineum1	147	10104.9	23.65	0.953	F	5061.6	6313	7562.2	8332.7	8410.3	9177.3
Ecoccineum2	107	8306.9	24.33	0.953	F	4291.2	5392.2	6450.3	7235.1	7054.5	7803.5
Ecoccineum3	90	7739.2	27.87	0.953	F	3947.1	5052.1	5852.5	6708.4	6486.3	7232
Ecordifolia1	162	9693.3	16.54	0.953	F	5311.4	6600.7	7956	8730.6	8604.1	9385
Fcupressoides1	134	5978.7	19.62	0.953	F	4798.7	6095.3	7204.6	8024.2	7843.4	8616.9
Fcupressoides2	118	5620.4	23.55	0.953	F	4561.1	5621.6	6745.3	7577.9	7401	8198.8
Felastica1	88	7585.7	38.10	1.000	A	3898.5	5048.5	5893.2	6772.2	6482.3	7186.7
Felastica2	83	7160.6	42.00	1.000	A	3862.6	4946.2	5750.8	6601.2	6378.1	7090.5
Felastica3	88	7566.5	38.10	1.000	A	3987.7	5064.6	5907.8	6765.2	6525.7	7192.5
Gavellana1	70	6516.6	28.05	0.953	F	3464.1	4511.4	5112.6	5953.3	5590.4	6432.3
Gavellana2	78	7005.8	29.22	0.953	F	3661.9	4682.9	5426.8	6253.1	6011	6788.2
Grobusta1	215	11771.6	25.30	1.000	A	6206.5	7679.2	9424.5	10209.6	10314.8	10982.7

Grobusta2	227	11938.6	24.90	1.000	A	6428.6	7938.8	9650.3	10472.1	10622.6	11279.7
Grobusta3	264	12717.1	26.60	1.000	A	6900.2	8464.2	10448.8	11222.1	11769.2	12411.4
Hhelix1	158	10339.1	30.50	1.000	A	5343	6583.7	8020.3	8842.6	8877.3	9539.5
Hhelix2	143	9763.3	31.80	1.000	A	5065.2	6317.4	7611	8446.3	8441.6	9057
Hserratifolia1	105	8043.0	28.57	0.953	F	4273.6	5364.6	6366.9	7184.3	7089.4	7842.6
Hserratifolia2	130	9009.2	28.54	0.953	F	4740.7	5957.3	7084.8	7895.8	8004.2	8766.1
Hserratifolia3	107	8150.0	31.56	0.953	F	4282	5391.4	6390.6	7220	7248.6	7980.6
Hydrangea1	108	7149.2	21.38	0.953	F	4317.2	5454.2	6446.2	7266.6	6988.6	7820.2
Hydrangea2	95	7610.3	23.46	0.953	F	4054.3	5163.7	6006.4	6849.6	6566.8	7345.9
Igermanica1	49	5835.9	28.46	1.000	A	2940.3	3938.4	4286.1	5205.6	4488.1	5285.3
Igermanica2	45	5203.4	42.18	1.000	A	2845.4	3824.4	4113.4	5028.2	4417.6	5206.1
Igermanica3	48	5105.0	39.51	1.000	A	2920	3920.6	4279.5	5154	4537.4	5342.9
Jchilensis1	182	9711.5	25.38	1.000	A	5660.1	7043.7	8605.4	9455.1	9391.9	10051.8
Jchilensis2	161	9692.7	27.90	1.000	A	5355.3	6649.8	8083.6	8914.6	8842.3	9522.3
Jchilensis3	157	9421.6	25.82	1.000	A	5335.6	6606	7984.5	8835.8	8640.7	9313.5
Lapiculata1	226	11437.2	22.52	0.953	F	6268.8	7714.5	9442.7	10195.1	10653.8	11400
Lapiculata1	503	18244.0	22.60	1.000	A	9607.9	11660.9	14420.6	15235.6	16967.9	17487.2
Lapiculata2	406	16082.6	23.70	1.000	A	8617	10565.2	12957.3	13746.3	15004	15524.7
Lapiculata3	401	15889.7	25.20	1.000	A	8545.2	10401	12884.5	13680.6	15097.8	15663.9
Lhirsuta1	128	9301.2	27.05	0.953	F	4694.1	5910.4	7040.7	7875.1	7886.5	8652.2
Lhirsuta2	124	9079.2	27.49	0.953	F	4631.1	5800.7	6922.7	7718.6	7755	8489.7
Lhirsuta3	122	8970.7	27.49	0.953	F	4598.7	5779.7	6845.2	7651	7679	8400.8
Lphilippiana1	44	4981.2	37.32	0.953	F	2728	3675.5	3985.8	4834.2	4358.5	5224.1
Lphilippiana2	43	5050.0	35.22	0.953	F	2750.1	3663.9	3902.7	4791.9	4300	5142
Lphilippiana3	43	5175.3	17.80	0.953	F	2697.1	3661.7	3938.2	4791.4	4152.3	5085.1
Lradicans1	72	5326.8	17.80	0.953	F	3535	4559.4	5196.1	6057.8	5560.6	6413.9

Lradicans2	45	2990.9	16.24	0.953	F	2808.7	3746.9	4028.3	4899.6	4253	5220.8
Lradicans3	68	4720.0	11.56	0.953	F	3434.7	4414.3	5039	5907	5305.7	6244
Lrosea1	22	3618.2	21.77	0.953	F	1980.5	2830.7	2651.7	3607.1	2781.4	3816.1
Lsempervirens1	37	4860.1	38.19	0.953	F	2562.8	3449.4	3606.7	4472.6	3943.6	4842.9
Lsempervirens2	29	4385.8	41.70	0.953	F	2254.6	3148.1	3115.7	4061	3449.9	4376.6
Lsempervirens3	31	4282.7	42.93	0.953	F	2322.3	3220.2	3224.6	4159.3	3564.7	4508.3
Mboaria1	181	10714.9	30.76	0.953	F	5642.8	6964.1	8395.4	9199.9	9859.3	10523.7
Mboaria2	127	8940.6	26.97	0.953	F	4667.3	5870.1	6975.8	7807.1	7848.2	8585.1
Mboaria3	163	10522.1	27.16	0.953	F	5317.5	6575.5	7976.4	8734.3	9053.6	9792.4
Mboaria1	168	10665.8	23.10	1.000	A	5503.6	6818.5	8282.7	9076.3	8851.9	9551.4
Mboaria2	169	10690.6	22.80	1.000	A	5539.2	6816.8	8306.5	9126.5	8829	9534.1
Mboaria3	159	10260.6	25.50	1.000	A	5319	6633.2	8039.5	8859.1	8680.1	9386.5
Mcoccinea1	84	6437.6	24.15	0.953	F	3841.1	4846.5	5608.7	6501.6	6170.4	6954.1
Mcoccinea2	57	4963.4	25.87	0.953	F	3129	4110.5	4581	5451.8	4942.6	5835.3
Mcoccinea3	75	5929.0	28.44	0.953	F	3594.6	4624.3	5310.6	6188.5	5869.8	6664.5
Mexsucca1	131	7168.8	16.42	0.953	F	4794	5950.8	7118.8	7921	7648.1	8484.8
Mexsucca2	117	6580.4	24.39	0.953	F	4472.4	5676.4	6682.9	7521.4	7416.3	8194.7
Mgrandiflora1	258	12967.2	26.30	1.000	A	6812.1	8349.3	10301.3	11108.1	11626.6	12197.8
Mgrandiflora2	236	12824.5	27.50	1.000	A	6514.9	8024.8	9844.1	10659.9	11081.4	11704
Mgrandiflora3	252	13112.0	20.90	1.000	A	6782	8291.2	10175.2	11002.2	11017.1	11658.3
Muehlenbeckia1	60	6310.3	29.71	0.953	F	3223.3	4206.5	4758.4	5567.9	5162.3	5996.8
Ndombeyii1	199	9398.3	23.36	0.953	F	5873.3	7257	8817.4	9595.1	9967.5	10674.6
Ndombeyii2	232	10828.2	25.74	0.953	F	6388.8	7827.5	9537.3	10308.9	11087.8	11715.6
Ndombeyii3	227	10177.3	25.70	0.953	F	6296.7	7769.8	9425.8	10244.8	10953	11572.3
Palqui_1	60	5871.5	31.10	1.000	A	3248	4318.9	4821.1	5707.5	5071.6	5856.2
Palqui_2	86	6470.7	35.40	1.000	A	3913.7	5020.1	5824.1	6709.9	6357.6	7048

Palqui_3	67	5988.4	30.10	1.000	A	3476.5	4511.8	5096.5	5953.7	5374.9	6152.5
Pandina1	85	5511.6	45.83	0.953	F	3859.1	4888.4	5684.3	6506.5	6741.5	7438.4
Pandina2	73	5509.4	46.28	0.953	F	3543.6	4603.8	5264.1	6055.3	6156.6	6831
Pboldus1	61	5707.4	34.23	0.953	F	3247.6	4207.5	4743.2	5629	5282.4	6067.6
Pboldus2	85	6456.7	35.86	0.953	F	3824.3	4854.9	5686.6	6512.8	6471.6	7183.4
Pboldus3	85	6763.1	36.96	0.953	F	3790.6	4874.1	5682.8	6530.2	6457.8	7234.6
Pcanariensis1	187	10519.3	28.14	1.000	A	5837.3	7144.6	8730.3	9540.6	9668.1	10358.2
Pcanariensis2	185	10761.5	21.21	1.000	A	5802	7193.5	8723.3	9531.7	9229.1	9987.8
Pcanariensis3	187	10342.1	29.93	1.000	A	5823.9	7124.5	8744.1	9524	9807.9	10412.5
Pdioicah1	167	8554.6	26.20	1.000	A	5503.8	6773.2	8278.6	9057.9	8951.4	9634.7
Pdioicah2	152	8403.5	23.20	1.000	A	5244	6496.1	7847.8	8642.4	8350.3	9069.1
Pdioicah3	183	9087.1	25.40	1.000	A	5697.5	7085.1	8668.9	9430.8	9433.3	10068.7
Pdioicam1	132	8440.3	18.20	1.000	A	4894.3	6071.2	7311.7	8137.1	7570.8	8330.7
Pdioicam2	124	7800.0	26.90	1.000	A	4708.2	5859.2	7093.2	7927	7558.3	8257.4
Pdioicam3	165	9088.1	27.20	1.000	A	5457.9	6753.1	8214.6	9031.9	8911.8	9619
Plingue1	174	9975.0	17.77	0.953	F	5487.1	6829.7	8186.6	9028.7	8996.4	9781.1
Plingue2	152	9030.7	17.76	0.953	F	5133.3	6341.6	7691.6	8476.3	8365.5	9150.3
Plingue3	191	10483.7	18.86	0.953	F	5796.2	7138	8628.9	9393.1	9513.5	10291
Pmagellanica1	108	7282.6	16.72	0.953	F	4288	5448.7	6431.1	7259.4	6941.6	7753.8
Pmagellanica2	84	6397.5	22.95	0.953	F	3819.5	4870.6	5641.2	6479.8	6145.1	6942.6
Pmagellanica3	109	7090.5	19.92	0.953	F	4330	5447	6467.9	7280.2	6953.8	7834.4
Pnubigenus1	125	8346.8	50.36	0.953	F	4629.3	5832.6	6933.5	7761.2	8841.1	9420.9
Pnubigenus2	146	9093.7	43.64	0.953	F	5044.5	6239.8	7513.6	8328.4	9388.7	9962.7
Pnubigenus3	163	9917.3	33.68	0.953	F	5287.5	6595.8	7940.5	8759.3	9426	10078.8
Pradiata1	49	4620.6	40.67	1.000	A	2926.2	3938.3	4275	5200.3	4580.6	5420.6
Pradiata2	42	4511.9	46.35	1.000	A	2747.6	3785.3	3937.5	4870.3	4289.3	5073.4

Pradiata3	60	6046.7	46.35	1.000	A	3283	4309.3	4814.4	5670.7	5337.2	6034.3
Psaligna1	100	7615.9	33.41	0.953	F	4140	5254.8	6146.7	7026	7028.3	7768.4
Psaligna2	93	6856.5	30.48	0.953	F	3986.1	5089	5944	6746.1	6628.9	7407.1
Psaligna3	88	7219.0	32.26	0.953	F	3914.5	4977	5776.7	6605.9	6458.7	7248.2
Qsaponaria1	178	10681.2	16.25	0.953	F	5521.5	6927.6	8343.7	9121.2	9021.1	9815.2
Qsaponaria2	121	8476.3	23.09	0.953	F	4566.9	5733.2	6840.7	7629.7	7492.4	8301
Qsaponaria1	207	12027.7	21.70	1.000	A	6141.4	7581.2	9220.7	10033	9869.5	10570.8
Qsaponaria2	208	12073.1	23.80	1.000	A	6150	7576.6	9239.6	10089.1	10016.6	10719
Qsaponaria3	215	12434.0	20.50	1.000	A	6208.7	7700.2	9401.8	10218.6	10016.2	10703.7
Rhododendron1	193	9943.3	24.52	0.953	F	5827.7	7156.5	8665.7	9487.3	9835.6	10561.5
Rhododendron2	189	9284.4	23.93	0.953	F	5751.6	7090.5	8592.3	9430.1	9699.4	10442
Rhododendron3	148	8036.1	22.75	0.953	F	5081.2	6263	7545.8	8411.2	8413	9147
Rhumilis1	187	10264.1	24.71	1.000	A	5830.6	7115.5	8763	9514.5	9467.3	10177.1
Rhumilis2	192	10128.9	25.11	1.000	A	5882.5	7227.1	8882.5	9680.8	9642.3	10323.5
Scassioides1	104	7552.0	15.76	0.953	F	4216.1	5382.1	6298.9	7159.7	6770.4	7606.4
Sconspicua1	171	8780.1	33.62	0.953	F	5448.4	6835.3	8180.9	8933.9	9701.9	10334.1
Sconspicua2	173	8622.7	34.21	0.953	F	5505.3	6792.1	8205.9	8973.9	9810	10436.3
Sscandens1	38	4577.7	41.15	0.953	F	2584.4	3452.8	3637.9	4546.3	4043.3	4940.1
Sscandens2	26	3823.3	41.15	0.953	F	2124.2	3003.8	2941.7	3876.3	3169.7	4107.9
Sscandens3	47	5413.8	38.06	0.953	F	2850.3	3791.9	4114.6	4986.9	4577.2	5422
Ssempervirens1	67	5568.9	56.64	0.953	F	3413.1	4418.5	4987.6	5850.9	6074.9	6711
Ssempervirens2	73	5737.0	56.52	0.953	F	3544.4	4566.1	5245.8	6093.5	6422	7062.2
Ssempervirens3	64	4639.2	55.67	0.953	F	3304	4343.4	4904.2	5732.3	5865.1	6554.3
Ssempervirens1	110	6903.7	55.98	1.000	A	4447.6	5604.3	6651.3	7470.2	8271.4	8767.4
Ssempervirens2	94	6260.2	54.96	1.000	A	4101.7	5193.7	6097.7	6958.7	7393	7977.8
Ssempervirens3	93	5934.6	48.65	1.000	A	4076.1	5188.4	6042.5	6960	7071.9	7710.1

Tcorymbosus1	36	4905.7	61.27	0.953	F	2498.8	3397.5	3565.9	4446.9	4135.2	4937.7
Tcorymbosus2	37	4854.0	61.24	0.953	F	2503.9	3477.6	3596.8	4519.7	4238.2	5041.8
Tcorymbosus3	41	5201.0	52.93	0.953	F	2667.7	3579	3839.8	4714.9	4407.4	5170.7
Tstipularis1	321	11645.7	22.45	0.953	F	7495.8	9158.3	11253	12039.1	13084.1	13724.3
Uncinia1	72	6002.2	28.71	0.953	F	3491.3	4518.1	5204.9	6039.4	5669.3	6526.2
